# Rates of return to sorghum and millet research investments: A meta-analysis

**DOI:** 10.1371/journal.pone.0180414

**Published:** 2017-07-07

**Authors:** Yacob A. Zereyesus, Timothy J. Dalton

**Affiliations:** Department of Agricultural Economics, Kansas State University, Manhattan, Kansas, United States of America; International Nutrition Inc, UNITED STATES

## Abstract

Sorghum and millet grow in some of the most heterogeneous and austere agroecologies around the world. These crops are amongst the top five cereal sources of food and feed. Yet, few studies document the impact of sorghum and millet genetic enhancement. The Internal Rate of Return (ROR) is one of the most popular metrics used to measure the economic return on investment on agricultural research and development (R&D). This study conducted a meta-analysis of 59 sorghum and millet ROR estimates obtained from 25 sources published between 1958 and 2015. The average rate of return to sorghum and millet R&D investment is between 54–76 percent per year. All studies computed social rather than private RORs because the technologies were developed using public funds originating from host country National Agricultural Research Systems (NARS) and international organizations such as the INTSORMIL CRSP, ICRISAT and others. Nearly three quarter of the studies focused only on sorghum (72 percent) and around one tenth of the studies (8 percent) on millet. Regression models analyzed the determinants of variation in the reported RORs. Results show that ex-ante type and self-evaluated type of analyses are positively and significantly associated with the ROR estimates. Compared to estimates conducted by a university, results from international institutions and other mixed organizations provided significantly smaller estimates. Estimates conducted at national level also are significantly lower than those conducted at sub-national levels. The ROR is higher for studies conducted in the United States and for those conducted more recently. The study also reconstructed modified internal rate of return (MIRR) for a sub-sample of the reported RORs following recent methods from the literature. These results show that the MIRR estimates are significantly smaller than the reported ROR estimates. Both results indicate that investment in sorghum and millet research generates high social rates of return.

## Introduction

Sorghum and millet are some of the world’s most important cereal crops especially in semi-arid areas of the Americas, sub-Saharan Africa and Asia. These two crops are important food and feed crops, especially in environments with heat and water stresses that characterize the semi-arid and arid drylands that are the home to 1.4 billion of the world’s population. A sizable portion of the world sorghum and millet production comes from sub-Saharan Africa and Asia [1]. For example, in 2014, of the top 20 sorghum and millet producing countries, 70 percent for sorghum and 85 percent for millet came from Africa and Asia. Three out of the top five highest sorghum-producing countries and the five most important highest millet-producing countries are from Sub-Saharan Africa and Asia [1]. Other major countries involved in sorghum and millet production and trade include the United States, Mexico, Argentina, Brazil, and Australia.

Even though the trends in total global acreage allocated to sorghum and millet have declined in recent years, the yield productivities (tons/ha) for both crops have been rising [1]. Investment in agricultural research and development (R&D) is likely to have contributed to the increased productivity growth since any opportunistic areal expansion is occurring on poor quality soils with limited productive capacity. Agricultural R&D is one key to productivity growth for agriculture and economic development yet many argue that there is weak evidence of the returns to research investment in dryland crops outside of major commodities of maize and wheat. To secure continuous funding for agricultural R&D, the return on such investment must be justified using social, economic, and environmental metrics. Research organizations, managers, funding institutions are increasingly allocating resources for conducting such impact assessment exercises. A number of studies have documented the overall impact of agricultural R&D investments (e.g. [2]; [3]; [4]). Few studies document the impact of sorghum and millet genetic enhancement. No specific global reviews of sorghum and millet exist in the literature. The current study is a systematic review and analysis of the economic impact of agricultural R &D investments on sorghum and millet. A review of the past studies of economic impacts of agricultural R&D investment on sorghum and millet will help provide an empirical support to the extent of the economic gains achieved from these two crops.

The Internal Rate of Return (commonly referred to as ROR) is one of the most popular metrics used to measure the economic return on investment to agricultural R&D. Based on a global compilation of data, Hurley, Pardey, Rao and Andrade (2016) reported that 94% of the reviewed studies report RORs, 34% report benefit-cost ratios (BCRs), and 25% report both as measures of outcome to investment in R&D [5]. Pardey, Andrade, Hurley, Rao and Leibenberg (2016) also report that based on a review of 113 studies conducted in sub-Saharan Africa, 74.3% reported only RORs, 10.7% reported on BCRs, and 15.9% report both RORs and BCRs[4]. Past literature indicates that the ROR is the preferred measure of outcome for agricultural R&D. The current study conducted a meta-analysis of 59 sorghum and millet reporting ROR estimates by compiling 25 studies. The average ROR to sorghum and millet R&D investment is in the range of 54–76 percent per year. All of the reviewed studies computed social rather than private RORs because sorghum and millet technologies were developed using public funds from host country National Agricultural Research Systems (NARS) and international partners such as the International Crops Research Institute for the Semi-Arid Tropics (ICRISAT), International Sorghum and Millet Collaborative Research Support Program (INTSORMIL CRSP), and others. Nearly three quarter of the studies focused only on sorghum (72 percent of the publications) and around one tenth of the studies (8 percent of the publications) dealt only with millet. Regression models analyze the determinants of variations in the reported RORs with various characteristics used as controls. Results show that ex-ante type and self-evaluated type of analyses are positively and significantly associated with the rate of return estimates. Compared to estimates conducted by a university-based scientist, results from international institutions and other mixed organizations provided significantly smaller estimates. The ROR is higher for studies conducted in the United States and for those conducted more recently. The study also reconstructed modified internal rate of return (MIRR) for a sub-sample of the reported RORs following recent methods from the literature [2]. These results show that the MIRR estimates are considerably smaller than the ROR estimates.

The rest of the study is organized as follows: The next section provides a description of the data and methods used to assemble the data. Then, the modeling section develops the functional relationship between reported RORs and its determinants and the approach followed to reconstruct the MIRR. The results section presents both descriptive and empirical results from the estimation models. The last section summarizes the study by providing the main conclusions and recommendations.

## Materials and methods

### Literature search

**Fig 1:**
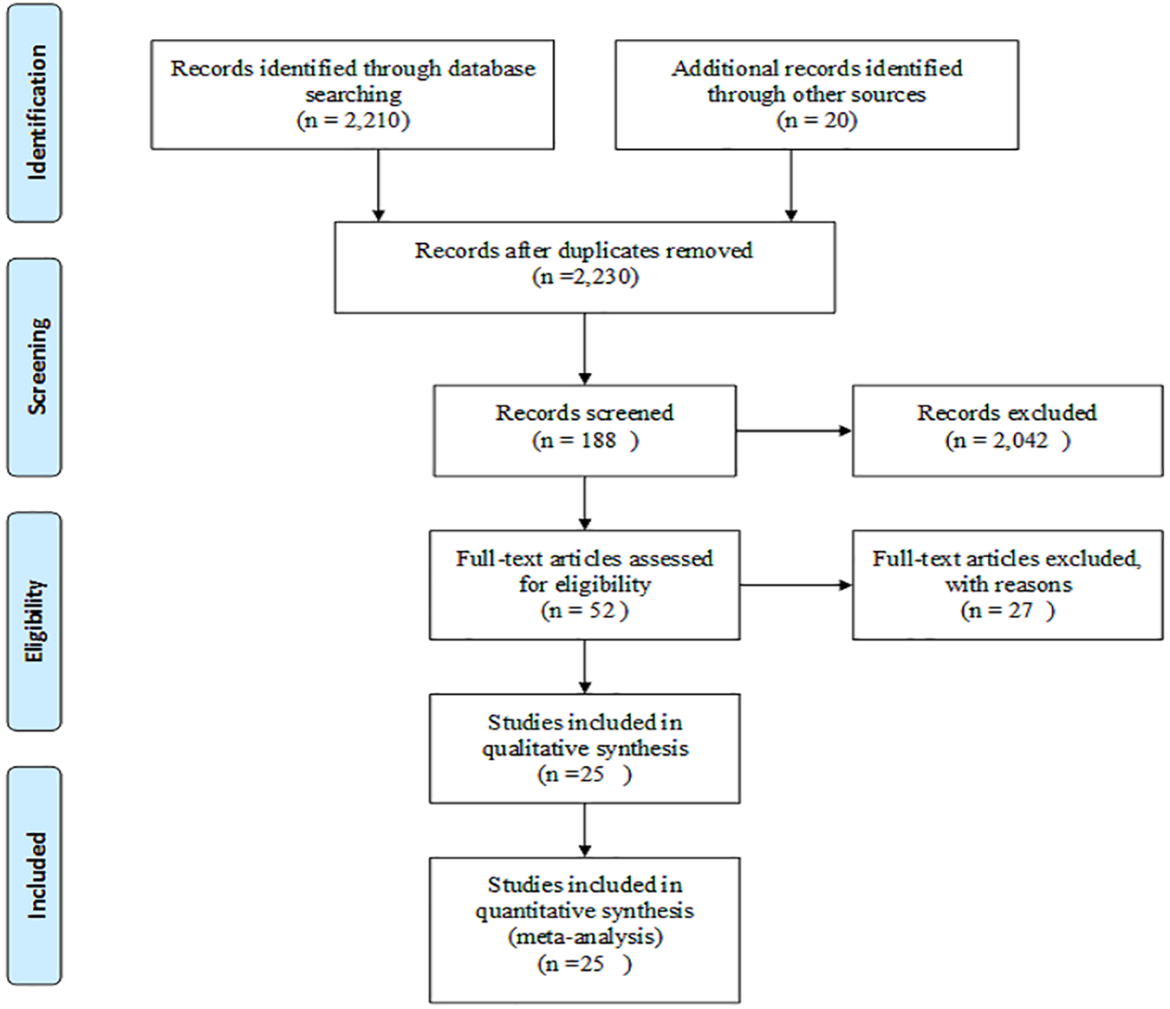
Flow diagram of literature search and screening process.

To identify the studies for the review, a comprehensive search compiled all the available evidence on the returns to agricultural R&D investments on sorghum and millet between 1958 and 2015. Online search engines such as Google Scholar, economic literature databases such as EconLit, AgEconSearch and JSTOR, Agricola etc. and the Consultative Group on International Agricultural Research (CGIAR)’s Standing Panel on Impact Assessment publications database were used for the search. Key word searches were applied in these search engines. The keyword search applied a combination of the following words or phrases: "Rate of return" “sorghum” “millet” "research and development”, "impact assessment”, "cost benefit”, "ex-ante" and "ex-post". The search initiated with the review of the abstract, the full text, and the reference sections of the latest publications available online or on print. Each of the references cited was reviewed for information on impact assessment studies on sorghum and millet. Based on this information, the relevant impact assessment studies on sorghum and millet are traced repeatedly until no more relevant reference citation is found on the reference sections in a systematic process that is generally referred to as “snowballing”. This structured search was complemented with direct personal contacts via email and phone calls with some of the authors to retrieve a few of the relevant studies since the base of literature covers several decades when manuscripts where not digitally available. In total, 25 studies and 59 ROR point estimates were assembled and are provided in **S1 Table**. A flow diagram showing the process of identification, screening, eligibility, and studies included is shown in Fig 1. Each study estimates a single rate of return for a scenario which is dependent upon data on adoption rates, prices and the shape of the supply and demand functions. While each of these elements affects the results of the meta-analysis, we do not have complete data on all assumptions in order to test their correlation with the outcome variable.

The reviewed studies included published reports (those that appeared in peer-reviewed journals, book chapters, evaluation and impact assessment reports) and unpublished reports (such as thesis, dissertations, or other gray reports). Guidelines on best-practice for conducting meta-regression analyses in the areas of research questions and effect size, literature searching, compilation and coding, and modeling issues were followed to improve the transparency and quality of the meta-analysis exercise [6].

### Statistical analysis

The primary meta-analysis focuses on the average ROR estimates disaggregated by various variables of interest. Most meta-analyses use impact variances or sample sizes as weights [7] if information about the variances and/or sample sizes used in each of the studies reviewed are readily available. Klumper and Qaim [8] used an alternative weighting procedure, the inverse of the number of outcome observations per data set, to test the robustness of their meta-analysis results. In our dataset, more than half (thirteen) of the studies had only a point estimate, four studies had two estimates, and four studies had three estimates. In total, 21 studies had three or fewer observations per study. Thus, it is not expected that any one study unduly dominate or bias the outcome effects. Similarly, forest plots could not be applied to our dataset because constructing forest plot requires information on the effect size (ROR in our case), lower confidence interval and upper confidence interval, which would not be meaningful in the current study because the majority of the studies included in the analysis are of a deterministic nature (i.e. a point estimate). However, a robust functional relationship is developed to explain the variability among the estimated outcomes values.

A functional relationship (*f*) can be developed to relate the rate of return measure (*m*) and its determinants as [2]:

m=m*(r)+v(a,r,e,u)=f(a,r,e)+u, (1)

where the vectors **m** refers to the rate of return measure while **a**, **r**, and **e** are the vectors of explanatory characteristics of the analysts performing the evaluation; the characteristics of the research being evaluated; and the features of the evaluation, respectively. The measure *m* is equal to the true value of what was being evaluated m* and the additive measurement error *v*. The true measure m* depends only on the characteristics of the research being evaluated (r), whereas the measurement error *v* depends on the same characteristics of the research but also on various other explanatory factors, as well as the truly random component *u*. The current study included variables relevant to sorghum and millet commodities. Alston, Chan-Kang, Marra, Pardey and Wyatt. [2] present a comprehensive description of the model and variables to be included in the meta-analyses.

### Modified internal rate of return

In spite of the wide application and popularity of ROR estimates, there has been growing skepticism about the reliability of previous reported RORs (e.g. [9]). The majority of studies based on ROR estimates of investments are hypothesized to systematically overstate the returns to agricultural R&D.

An alternative to the traditional internal ROR is the Modified Internal Rate of Return (MIRR). The MIRR, which is a derivative of the ROR, provides a more accurate percentage measure of financial attractiveness and it addresses the two shortcomings of the ROR. The MIRR implicitly relaxes the assumption that an investment’s rate of return is equal to the reinvestment and cost of capital rates, and that the MIRR is unique ([10], [11]). The MIRR is also considered to have strong theoretical base [12]. When evaluating public investments in R&D, conceptually the MIRR is superior to the traditional internal ROR [9]. Empirical evaluation of MIRR is facilitated if data on the term of the investment, stream of benefits and costs, and the reinvestment and cost of capital discount rates are readily available. Most studies on agricultural R&D in the past do not report the stream of benefits and costs. Hurley, Rao and Pardey [5]developed an approach to recalibrate the estimated historical rates of return in agricultural R&D and reconstructed the MIRR using a comprehensive ROR studies published between 1958 and 2011.

The MIRR is defined as $\sqrt[{T}]{\frac{FVB}{PVC}}-1$ where T > 0 is the term of the investment, FVB ≥ 0 is the future value of the investment benefits, and PVC ≥ 0 is the present value of the investment costs. The discount (reinvestment) rate used to compute FVB and the discount rate (or cost of capital) used to compute PVC need not be equal to each other in the MIRR, unlike the standard ROR which implicitly assumes that the investment’s rate of return is equal to the reinvestment and cost of capital rates. To reconstruct the MIRR, Hurley, Rao and Pardey [11] begin with the relationship between MIRR and Benefit Cost Ratio (BCR) as noted by Anthanasopoulos [13] and Negrete [14]. This relationship is:

$$MIRR=(1+\delta )\sqrt[{T}]{BCR}-1$$ (2)

Where *δ* is the discount rate used to evaluate the BCR and T is the term of investment. Hurley, Rao and Pardey [11] introduce differing discount rates into the previous equation such that the investment’s rate of return need not be equal to the reinvestment and cost of capital rates. This was done using the following general formula:

$$MIRR=\sqrt[{T}]{BCR\frac{\sum_{t=0}^{T}w_{c_{t}}{\left(1+\delta \right)}^{-t}}{\sum_{t=0}^{T}w_{b_{t}}{\left(1+\delta \right)}^{-t}}\frac{\sum_{t=0}^{T}w_{b_{t}}{\left(1+\delta^{r}\right)}^{T-t}}{\sum_{t=0}^{T}w_{c_{t}}{\left(1+\delta^{c}\right)}^{-t}}}-1$$ (3)

Where $w_{c_{t}}$ and $w_{b_{t}}$ are the proportion of the total undiscounted costs and benefits accruing at time *t*, and *δ*^*r*^ and *δ*^*c*^ are the reinvestment and cost of capital rates. When *δ* = *δ*^*r*^ = *δ*^*c*^, then Eq 3 reduces to Eq 2. To solve MIRR in the above equation, data are required on the term of investment (T), BCR and the associated discount rate, the distribution of costs and benefits, and the reinvestment and cost of capital rates. Using only information on the term of investment (T), BCR, and *δ*, Hurley, Rao and Pardey [11] reconstructed the information on the distribution of costs and benefits. This was done by further manipulating the relationship between BCR and ROR that also depend on *δ* and the distribution of costs and benefits as:

$$BCR=\frac{\sum_{t=0}^{T}w_{b_{t}}{\left(1+\delta \right)}^{-t}}{\sum_{t=0}^{T}w_{c_{t}}{\left(1+\delta \right)}^{-t}}\frac{\sum_{t=0}^{T}w_{c_{t}}{\left(1+ROR\right)}^{-t}}{\sum_{t=T_{b}}^{T}w_{b_{t}}{\left(1+ROR\right)}^{-t}}$$ (4)

This equation calls for the identification of distributions that satisfy the stated relationship and these distributions substituted into Eq (3) with any desired reinvestment and cost of capital discount rates to calculate the MIRR.

Hurley, Rao and Pardey [11] assume a two-parameter, unit trapezoidal distribution to reconstruct the distribution of the costs and benefits. The use of the two-parameter, unit trapezoidal characterization of costs and benefits makes it possible to easily search for the distributions that are closest to Eq (4). The individual MIRR values for the reported ROR estimates of sorghum and millet studies used in the current paper were reconstructed following the derivations and the grid search discussed in the research paper by Hurley, Pardey, Rao and Andrade [5]. To calculate the MIRR in the current paper, a discount rate of 3 percent and a reinvestment rate of 3.5 percent were assumed following Hurley, Rao and Pardey [11] who use of 3 percent cost of capital rate was based on the average rate of return for long-term U.S. treasuries and a reinvestment rate of 3.5 percent which is the average of the average rate of return to long-term U.S. treasuries and Standard & Poor’s 500 equity index from 1969 to 2010 to explore the implications of recalibrating internal ROR. The minimum information required to do the grid search was available only for 24 observations.

## Results and discussion

### Effect sizes of rates of return

**Fig 2:**
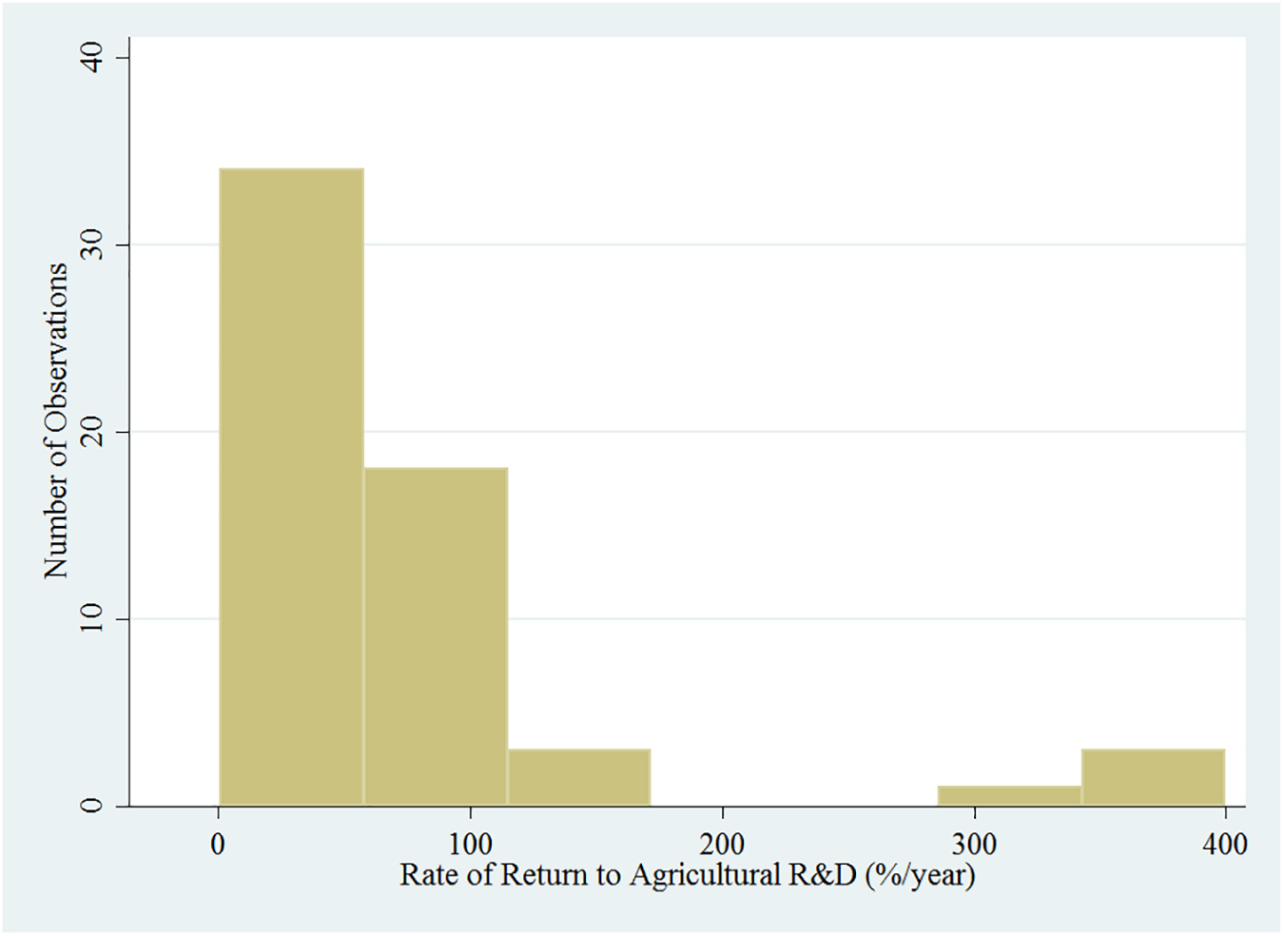
Distribution of the rates of return to agricultural research and development on sorghum and millet over all studies.

**Fig 3:**
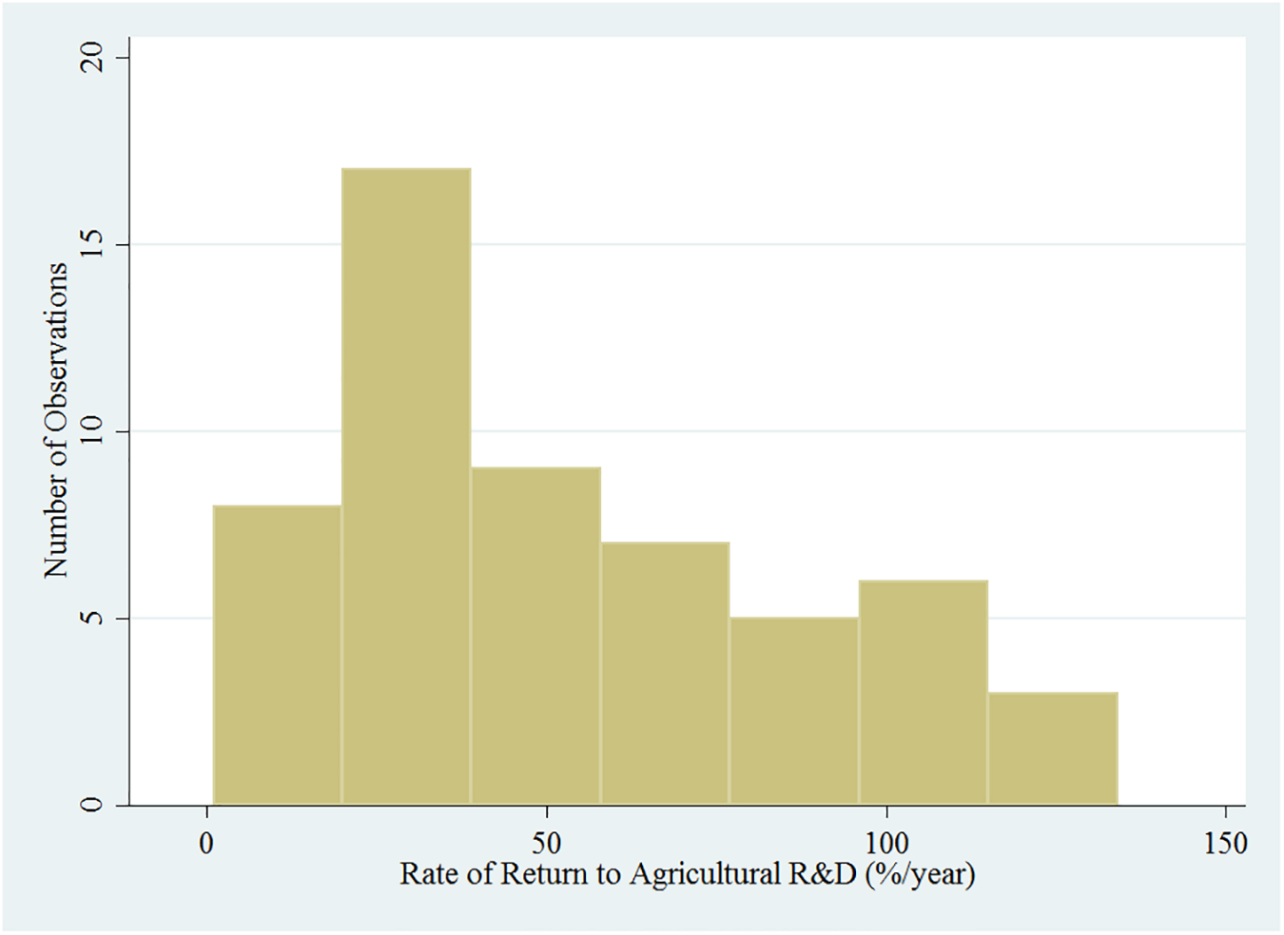
Distribution of rates of return to agricultural R&D on sorghum and millet excluding extreme values.

Over all, there are 25 publications and 59 ROR point estimates collected from the studies. All except one of the studies computed the ROR estimates. The distribution of the ROR to sorghum and millet for all the observations appears to have a bimodal distribution (Fig 2). The mean and median ROR for this set of data were 75.6 percent and 48.2 percent, respectively. Two publications (4 point estimates) have reported RORs close to 400 percent. These relatively high RORs, which were conducted in the United States, may be due to the better technology packages available in the United States that facilitate the adoption and diffusion of the technologies much more quickly compared to other less developed countries, or spill-in benefits from international research. The distribution of the RORs excluding these two publications is shown in Fig 3. The mean and median ROR estimates for this set of data were 54.0 percent and 43.0 percent, respectively. There is high dispersion of the observations around the mean for the data set in Fig 2, with standard deviation of 88.5, compared to the data set in Fig 3 with a much smaller standard deviation of 37.0. In a meta-analysis of ROR to agricultural R&D, Alston, Chan-Kang, Marra, Pardey and Wyatt [2] report that the mean internal ROR for a sample of 1,852 estimates was 81.3 percent per year. After removing outliers and incomplete observations, they found that the mean internal ROR for a sample of 1,128 estimates was 64.6 percent per year. Alston, Chan-Kang, Marra, Pardey and Wyatt [2] concluded that in general agricultural R&D has paid off “handsomely” for society. Focusing on the main U.S. public agricultural research institutions, Huffman [15], Huffman and Evenson [16], Huffman, Norton and Tweeten [17] document a 50 percent marginal rate of return from agricultural productivity. Without excluding the very high ROR estimated values, the data was log transformed and plotted as shown in S1 Fig. The mean and median values of this transformed data are 3.8 and 3.9, respectively.

### Characteristics of the rate of return measure (*m*)

Variables related to the rate of return (*m*) may include whether the ROR measure was estimated in *real or nominal* financial values, *marginal or average* ROR, and whether the estimate is in *social* or *private* terms. These variables could potentially contribute to the systematic variation in the RORs across the studies. Ideally, different measures would be segregated and analyzed independently but because the sample of sorghum and millet studies is limited in size they are pooled and controlled for with an explanatory variable. Among these variables, only the *ex-ante or ex-post* variable is used because there was insufficient variation in the other variables (e.g. all but one of the studies computed social rates of return) and because of lack of adequate information in the case studies reviewed (e.g. real versus marginal). The majority of the studies were ex-post type of analyses (60 percent of the publications and 63 percent of the point estimates) indicating that most of the ROR studies on sorghum and millet were conducted to evaluate the impact of past R&D investments. One study applied both ex-ante and ex-post types of analyses. If we look at the African continent, all except one study were an ex-post type of analyses (Table 1). Most of the studies (88 percent of the publications and 80 percent of the point estimates) computed an average RORs compared to marginal RORs. This is because of the widespread use of the economic surplus method to evaluate the benefits of R&D to society. In addition, all of the studies reviewed computed social (as opposed to private) RORs (Table 1). This is particularly true in the African case studies, because all of the technologies developed used public funds from the national agricultural research systems (NARS) and from international partners such as the International Sorghum and Millet collaborative research support program (INTSORMIL CRSP), International Crops Research Institute for the Semi-Arid Tropics (ICRISAT) and other collaborating public institutions.

**Table 1:** Descriptive statistics on sorghum and millet rate of return studies.

Characteristic	Number		Share of respective total	
	Publications	Estimates	Publications	Estimates
**Ex-ante or ex-post rate of return**	(count)		(percentage)	
Ex-ante	6	13	24	22
Ex-post	15	37	60	63
Ex-ante/ Ex-post	1	6	4	10
Unclear	3	3	12	5
Total	25	59	100	100
**Average or marginal rate of return**			
Average	22	47	88	80
Marginal	1	10	4	17
Unclear	2	2	8	3
Total	25	59	100	100
**Private or social rate of return**				
Private	0	0	0	0
Social	23	57	92	97
Unclear	2	2	8	3
Total	25	59	100	100
**Geographic Region**				
Africa	15	28	60	47
United States	5	24	20	41
Central America	2	2	8	3
India	1	3	4	5
Unclear	2	2	8	3
Total	25	59	100	100
**Institutional Sources of Technology for Sorghum and Millet**					
INTSORMIL only	3	9	12	15
ICRISAT only	9	13	36	22
Both INTSORMIL and ICRISAT	3	5	12	8
Other	9	30	36	51
Unclear	1	2	4	3
Total	25	59	100	100
**Commodity of Analysis**				
Sorghum	18	45	72	76
Millet	2	6	8	10
Both	4	7	16	12
Unclear	1	1	4	2
Total	25	59	100	100

#### Analyst characteristics (a)

Given that a significant amount of the R&D investment on sorghum and millet is affiliated with specific organizations and institutions, the question of whether or not the evaluation of R&D work represents a *self-evaluation* forms an important factor that may tend to affect the results on the rates of return favorably or unfavorably. For example, Alston, Chan-Kang, Marra, Pardey and Wyatt [2] explain that, in many cases, the rate of return to research expenditures is estimated by researchers associated in some way with the research or the research institution being evaluated. They contend that self-evaluation could possibly introduce upward bias in the estimate due to self-interest but this could be offset by a self-evaluator who may better understand the research being evaluated or who might have better access to data and other information. Nonetheless, the direction of any such effect is unclear.

Some variation among studies may be associated with variations among individuals in what they work on, how they go about their work, and what procedures they use. Three categories of variables were developed to refer to the research organization conducting the impact assessment study: 1) Universities; 2) International institutions (ICRISAT, ILRI, FAO, NARS) and 3) Mixed which is a combination of these groups. Since a significant amount of sorghum and millet R&D is concentrated in a very limited number of research organization such as the INTSORMIL CRSP, ICRISAT, or universities, this variable may capture biases but the sign is derived empirically, although skeptically hypothesized as positive.

Whether or not the research work is published as a peer-reviewed manuscript may also have a bearing on the result of the rate of return. Alston, Chan-Kang, Marra, Pardey and Wyatt [2] note that this aspect reflects the types of reviewer scrutiny to which the work was subjected, but the prepublication review process may also discriminate against studies that generate rates of return that fall outside the range of “conventional wisdom” prevailing in the profession at the time or that it may not be desirable to publish.

#### Research characteristics (r)

The rate of return is likely to vary with nature of the characteristics of the research itself, for example, whether it focuses on genetic enhancement, natural resource management, crop management or extension [18]. The current study benefits by controlling for the sources of variation that are associated with the research characteristics, for example the need to classify the studies by commodity. Because this study focuses on sorghum and millet only, there is no need to classify the ROR studies by commodity classes. Due to inadequate number of observations, estimates were aggregated across fields of science *(basic*, *applied*, *and extension)* and the type of technology *(yield enhancement*, *pest or disease control*, *crop management*, *and extension)* despite that the majority of studies were on crop genetic improvement. It was not possible to extract information for all the studies on the period when the research was evaluated, for example in early adoption or mature state, or the narrow geographical region where the R&D was conducted and the geographical region where the results were adopted beyond national levels. The publication date of the study and the studies are divided into those that occurred prior to the median study date and those after. The sign on this is ambiguous as it could be argued that “low hanging fruit” were first addressed and hence earlier studies may have generated higher returns. Conversely, more recent technologies could be better refined and more productive. The *Scope of research* variable captures the geographic coverage of the study. These were grouped into 1) sub-national—if the study covers only one region or area or state inside a country; 2) national- if the study was conducted at a country level; and 3) multinational- if the study extended to multiple countries such as regional economic blocs (e.g. SADC in the Southern Africa).

Sorghum and millet grow in very harsh environments where other cereal crops do not perform well. Millions of the poorest people in the semi-arid tropics of Africa and Asia consume sorghum and millets. In general, due to these production and peculiar consumption characteristics of sorghum and millet, the sorghum and millet ROR studies are confined to specific geographic regions of the world. Geographic variables denoting studies in the United States and Africa are created to capture region specific differences. More than half of the impact assessment studies (60 percent of the publications) were conducted in Sub-Saharan Africa, followed by almost a quarter of the studies being conducted in the United States (20 percent) and the remaining small percentage in Central America (8 percent) and India (4 percent) (Table 1).

We isolate differences between sorghum and millet studies through to determine whether species-specific effects exist. Nearly three quarters of the studies focused only on sorghum (72 percent of the publications) and around one tenth of the studies (8 percent of the publications) dealt only with millet (Table 1). This result may due to the economic importance and wide range usage of sorghum in the countries where the studies were conducted and the relatively higher proportion of investment expenses allocated by research institutions on sorghum over millet. We also isolate studies that measure the impact of new varieties that were generated by ICRISAT or the INTSORMIL CRSP to determine whether targeting technologies towards low-income countries produces results that are different from those in high-income countries. The hypothesized effects of both variables are ambiguous. More than half of the studies (60 percent of the publications) reported on the two major sorghum and millet improvement organizations around the world—the INTSORMIL CRSP (which has been succeeded by the Feed the Future Innovation Lab for Collaborative Research on Sorghum and Millet) and ICRISAT -as the primary source of the technology (e.g. germplasm to develop improved sorghum and millet varieties and hybrids). This is followed by the “Other” category (36 percent of the publications) such as universities that are not directly affiliated with these two institutions (Table 1).

#### Evaluation characteristics (e)

According to Alston, Chan-Kang, Marra, Pardey and Wyatt [2], several characteristics may affect the measurement of the research-induced change in yield, productivity, or the supply shift; others have implications on the size of measured benefits and costs of R&D for a given research-induced supply shift. Some factors include whether the study involves an explicit economic surplus analysis, with a formal supply and demand model, or whether it leaves the model implicit and uses an approximation based on a percentage research-induced supply shift multiplied by the initial value of production. The majority of the studies reviewed used an explicit economic surplus analysis, and so this set of variables may not be considered as a source of variation affecting the results of this study.

Studies that use explicit surplus measures require choices about the functional form of supply and demand (linear or constant elasticity) and the nature of the supply shift, that is whether it was either an Akino-Hayami pivotal (proportional) shift or a parallel (absolute) shift. Given the relative homogeneity in the use of explicit economic surplus analysis method in the current paper, the studies are categorized as parallel supply shifts, pivotal supply shifts, as well as “others” that do not fall under either of these two categories. The latter category does not assume anything about the supply shift; instead, they are composed of benefit- cost analyses.

### Meta regression results

Descriptive statistics of the variables used in the meta-analysis regressions is displayed in Table 2. The twelve estimates excluded from the meta-regression estimation include i) one study that used both ex-ante and ex-post type of analysis combined (6 estimates in total); ii) three estimates that did not have clear information whether the study was ex-ante or ex-post, and iii) three estimates that did not provide the publication status of the report. Almost 30 percent of the estimates are of an ex-ante and of self-evaluation type of reports. A quarter of the estimates were conducted by an analyst affiliated with an international institution and about 10 percent affiliated with an international and academic institution. Almost 70 percent of the analysts published the ROR estimates in journal articles. The median ROR reporting period is 1995. Forty-one percent of the estimated reports were published after the median reporting period. Only 4 percent of the research had a multinational coverage whereas three quarters of the research had a national coverage. About 40 percent of the ROR estimates were conducted in Sub-Saharan Africa and 41 percent of the estimates were conducted in the United States. Almost 4 in 5 of the estimates pertain to sorghum only study and 50 percent of the estimates germplasm sourced from either INTSORMIL or ICRISAT. Seventeen percent and twenty-three percent of the evaluations applied pivotal supply shift and parallel supply shift assumptions to estimates the RORs. Table 2 also presents a description of the conditional means of the rate of return for each of the dichotomous variables and the categorization. The first point of interest is that there are many variables with a limited number of observations in either of the categories. For example, there are 13 observations of ex-ante studies and 34 ex-post which does not permit a test of mean differences. Studies published on or after 1995 have a mean rate of return of 76.8% and studies published before the midpoint have the mean value of 62.7%.

**Table 2:** Descriptive statistics of the variables used in the regression models (N = 47).

			Mean ROR (%) conditioned by variable value	
	Mean	Std. Dev.	Yes = 1	No = 0
Ex-ante estimate	0.28	0.45	87	85
*Analysis characteristics*				
Self-evaluation	0.28	0.45	131	69
International institution affiliation	0.26	0.44	53	97
International and academic institution	0.09	0.28	11	93
Study published	0.68	0.47	95	66
*Research Characteristics*				
ROR reporting period**^**	0.41	0.50	99	65
Multinational scope	0.04	0.20	10	89
National scope	0.74	0.44	70	131
Sub-Saharan Africa region	0.38	0.49	40	114
United States region	0.51	0.51	130	39
Sorghum only	0.79	0.41	102	26
INTSORMIL or ICRISAT	0.49	0.51	87	85
*Evaluation characteristics±*				
Pivotal supply shift	0.17	0.38	28	98
Parallel supply shift	0.23	0.43	59	94

**^** the median ROR reporting period is 1995. The reporting period takes the value of 1 if the ROR report is published after the year 1995, and 0 otherwise.

*±* The ‘pivotal supply shift’ assumes a linear in logarithms supply function and shifts proportionally, whereas the “parallel supply shift’ assumes linear supply function and shifts in parallel.

Table 3 presents the result of five regressions that explain variation on the rate of return. A base regression is presented in column one and additional models in columns two to five. The regressions are estimated using robust standard errors, after it was revealed that some of the alternative models had some form of heteroscedasticity. The additional models test alternative specifications that emphasize the interplay between analyst, research and evaluation characteristics. Because there are limited observations, correlation between some variables exists and alternative specifications test the tradeoff between variables. These five models were also re-estimated by excluding the four very high ROR estimates as well as using the log transformed data set. Results from these alterative estimations provide similar implications and are presented in S2 and S3 Tables.

**Table 3:** Meta-analysis regression results for sorghum and millet ROR studies.

	ROR	ROR	ROR	ROR	ROR
Constant	133.00***	136.73**	145.52***	64.79	89.39**
	(4.04)	(2.71)	(3.13)	(1.24)	(2.59)
Ex-ante estimate	83.70**	25.86	-10.52	32.91*	15.00
	(2.22)	(1.31)	(-0.51)	(1.84)	(0.75)
*Analyst characteristics*					
Self-evaluation	221.53***				
	(6.39)				
International institution affiliation	-73.24*	-31.26		28.23	
	(-2.01)	(-1.00)		(0.97)	
International and academic institution	-56.62*	-205.99***	-228.15***	-210.46***	-231.61***
	(-1.91)	(-3.92)	(-3.78)	(-3.83)	(-3.53)
Study published	-37.59*	-3.03	9.53	-29.63*	-12.32
	(-1.81)	(-0.16)	(-0.61)	(-1.73)	(-0.77)
*Research Characteristics*					
ROR reporting period**^**		187.07***	188.64***	191.66***	192.88***
		(4.05)	(4.05)	(3.96)	(3.89)
Multinational scope	-110.34**	-61.58	17.36	-69.64	-32.34
	(-2.3)	(-1.45)	(0.32)	(-1.62)	(-0.5)
National scope	-74.95**	-77.38**	-84.83**	-78.14**	-78.42**
	(-2.49)	(-2.41)	(-2.27)	(-2.38)	(-2.39)
Sub-Saharan Africa region			-44.93**		
			(-2.57)		
United States region				94.02***	71.01***
				(6.65)	(3.13)
Sorghum only	27.93	12.22	30.02*	10.97	10.38
	(1.28)	(0.51)	(1.87)	(0.44)	(0.44)
INTSORMIL or ICRISAT			-37.38**		-19.82
			(-2.36)		(-1.02)
*Evaluation Characteristics±*					
Pivotal supply shift	-2.65	-69.55**	-30.58	29.84	8.32
	(-0.12)	(-2.28)	(-1.09)	(-0.85)	(-0.28)
Parallel supply shift	-186.06***	-214.33***	-201.86***	-178.45***	-172.95***
	(-4.39)	(-4.71)	(-6.11)	(-3.98)	(-4.21)
*R*^2^	0.70	0.83	0.88	0.88	0.88
*N*	47	46	46	46	46

* Significant at the 90 percent confidence level

** significant at the 95 percent confidence level

***significant at the 99 percent confidence level.

**^** the median ROR reporting period is 1995. The reporting period takes the value of 1 if the ROR report is published after the year 1995, and 0 otherwise.

*±* The ‘pivotal supply shift’ assumes a linear in logarithms supply function and shifts proportionally, whereas the “parallel supply shift’ assumes linear supply function and shifts in parallel.

Characteristics of the rate of return measure (*m*). The variable capturing whether the study was ex-ante or ex-post was statistically significant at 99% confidence level in the base model and in only two of the alternative models. Ex-ante studies have higher rates of return compared to ex-post studies. This finding is contrary to the Alston, Chan-Kang, Marra, Pardey and Wyatt [2] meta-analysis results. Using a larger data set across an extensive coverage of commodities and technologies, Alston, Chan-Kang, Marra, Pardey and Wyatt find that the returns on ex-post studies suggest the “cherry picking” of studies generating higher rates of return. A possible explanation for the findings of the present study may be associated with the particular regions of studies for sorghum and millet. The majority of the studies being ex-post type are conducted in the Sub-Saharan Africa. Evaluations of returns to research in such areas not only reflect the technology introduction, but also the cumulative effect of the market conditions and policy frameworks, which may in general reduce the potential economic impact of the introduced technology.

#### Analyst characteristics (a)

The variable on “self-evaluation” is statistically significant at the 99% level. Higher returns to research are associated when the study is self-evaluated rather than independent analysis, in contrast to the Alston, Chan-Kang, Marra, Pardey and Wyatt [2] findings. Studies published by individuals affiliated with universities are higher than those published by researchers from international organizations or teams composed of individuals from universities and international centers. The negative sign on the variables capturing mixed teams of researchers is consistent across all models. Published Studies (peer-reviewed journals, book chapters, evaluation and impact assessment reports) document lower rates of return than unpublished studies (thesis, dissertations, or any other unpublished gray reports) but this variable is only significant in two of the five models.

#### Research characteristics (r)

Several variables were significantly different from zero in the research characteristics area. Compared to the reference category of sub-national studies (if the study covers only one region, area or state inside a country), both national (nationwide) and multinational studies have lower rates of return. The explanation for these results may be related to the fact that aggregation over national and/or multinational data may possibly dilute the impact of the introduced technology and hence lower RORs compared to area-specific studies. It may also indicate that higher rates of return are generated when technology is narrowly targeted at a specific agroecology. The geographical specificity of research results is also apparent with variables that capture studies published on technologies generated for the United States and Africa, versus those targeting Latin America and Asia. In column three, a variable capturing studies focusing on research impact in sub-Saharan Africa is negative and significant while those published on U.S.-based research outputs are positive and significant (columns four and five). These variables are correlated with institutional affiliation of the author.

Alternative models include a variable that delineates the studies at the median year (“ROR reporting period”) to capture whether more recent studies are find higher rates of return than earlier studies. This variable is positive, significant and with a consistent magnitude in all of the alternative models. This variable is also correlated with the “self-evaluation” variable reflecting the increased demand for documented impact on agricultural investments and the self-sourcing of such studies.

Studies that focused on sorghum did not generate higher rates of return than millet-based studies except in one of the alternative models. This occurred in models where the source of germplasm was attributed to either one of two international-focused organizations: ICRISAT or INTSORMIL. This variable was not significant in other models especially where more variables controlling for the region are included. In addition, the variable on the INTSORMIL or ICRISAT source of germplasm was not statistically significant in the fifth model that includes a variable capturing U.S.-based research. Overall, there is correlation between the institutional affiliation of the researcher, the geographical focus of the technology and source of germplasm.

#### Evaluation characteristics (e)

According to the regression results, studies that assume a parallel supply shift generated lower rates of return compared to other supply shift assumptions. The parallel supply shift variable was negative, significant and consistent across all models. The default category (others) does not assume anything about the nature of the supply shift. Pivotal supply shifts were not statistically different from the reference variable. This is consistent with the fact that all of the studies conducted in the United States, with very high rates of return compared to other regions, used neither the pivotal nor the parallel supply shift assumptions.

### Modified internal rate of return results

The MIRR values were reconstructed for 24 observations with sufficient information to calculate the MIRR following the method discussed in the modeling section. The mean and median values for the reconstructed MIRR values were 22.4 and 20.1 percent, respectively. The correlation coefficient between the MIRR and ROR values for these 24 observations is 0.553. Using the MIRR estimates for these 24 estimates, an OLS regression is fitted to establish a relationship between the estimated MIRR and reported ROR. Based on the estimated regression relationship, predicted values for the rest of the observations are obtained. The mean value for the entire sample is 20.0, which is very close to the mean value obtained from the original 24 observations. Alston, Andersen, James, and Pardey [9] re-calculated the MIRR for investments on USDA agricultural R&D investments from 1949 to 2002. Assuming a 3 percent per year reinvestment and cost of capital rate, they found an average MIRR of 9.9 percent per year. Anderson and Song [19] also found that the average MIRR for US public agricultural R&D investment to be in the range of 8 to 10 percent per year.

## Conclusion

Historical returns on sorghum and millet R &D investments have been socially profitable. On a global coverage, the average ROR to sorghum and millet agricultural R&D investments is in the range of 58–81 percent per year. A number of notable results are observed from the review. The majority of the economic impact assessment studies are ex-post type analyses conducted in Sub-Saharan African countries. Both variables were negatively related to the rate of return. Self-evaluated impact assessment studies had higher RORs but this was counteracted if the self-evaluation was conducted by a researcher from an international institution or a team of researchers from universities collaborating with an international institution. Published studies presented lower rates of returns than unpublished manuscripts. Studies assuming a proportional supply shift produced results that were lower than others.

Studies that evaluated research impacts distributed over narrower geographical areas, as opposed to national or multinational impacts, reported higher rates of returns. This finding suggests that technological innovation should focus on narrow adaptation to agroecological conditions rather than panterritorial adaptation. In addition, we find that continental differences are important, with research in the United States generating the highest rates of return, and this may reflect the underlying economic environment and the cost of conducting research.

Investment rate of return estimates systematically overstate the returns to agricultural R&D. The study also reconstructed modified internal rate of return (MIRR) for a sub-sample of the reported RORs. These results show that the MIRR estimates are considerably smaller than the reported ROR estimates. It is important to note that the limited number of reported ROR estimates limits the generalization of the regression results of the study. The lack of variation in some of the determinants of the RORs also resulted in exclusion of some important variables. Nevertheless, the modified rates of return on sorghum and millet reinforce the message generated in the meta-analysis of the non-modified rates of return: the social returns to research investment on sorghum and millet are positive and high, at about 20% per year, and demonstrate that research is generating large producer and consumer benefits for billions of individuals located in the semi-arid and arid regions of the world.

## Supporting information

S1 Fig Distribution of the log of the rates of return to agricultural R&D for Sorghum and Millet for the entire studies. (TIF)

S2 Fig PRISMA checklist for meta-analyses. (DOC)

S1 Table List of publications used in the meta-analysis. (DOCX)

S2 Table Meta-analysis regression results for sorghum and millet ROR studies excluding very high ROR estimates. (DOCX)

S3 Table Meta-analysis regression results for sorghum and millet ROR studies with log transformed RORs. (DOCX)

S1 Data Data used for the meta-analysis. (XLS)
