# Supplementary figures and images for: Rates of return to sorghum and millet research investments: A meta-analysis
Source: PLoS One. 2017 Jul 7;12(7):e0180414. doi: 10.1371/journal.pone.0180414 (PMC5501525; doi:10.1371/journal.pone.0180414)

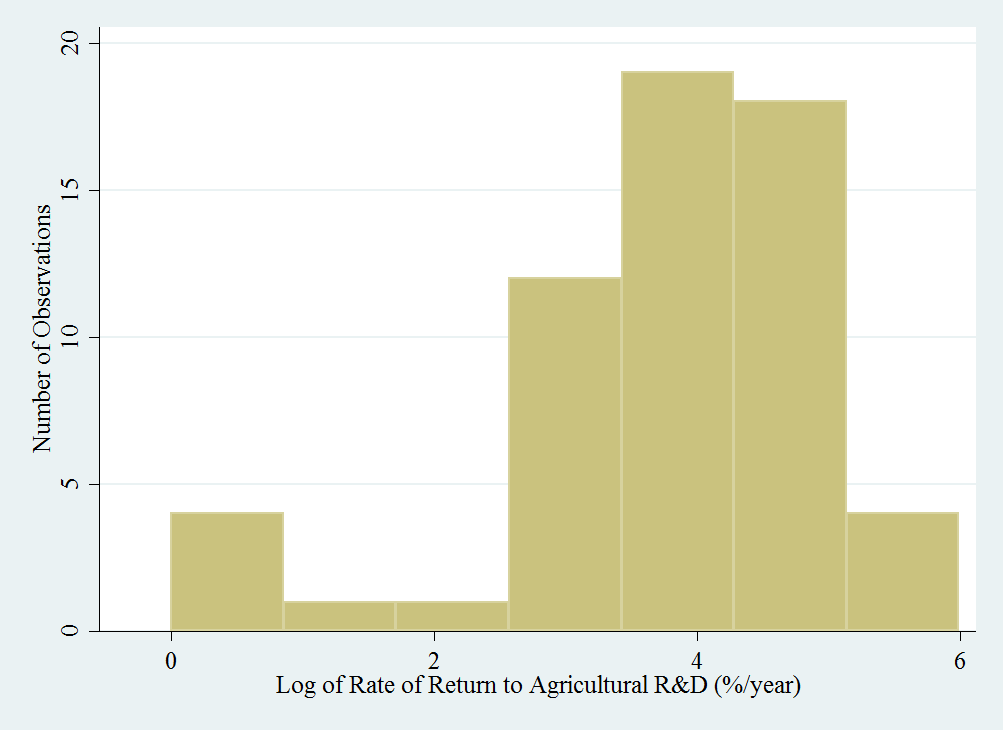

Supplement: S1 Fig — (TIF) [file pone.0180414.s001.tif]
