# Supplementary material for: Rates of return to sorghum and millet research investments: A meta-analysis
Source: PLoS One. 2017 Jul 7;12(7):e0180414. doi: 10.1371/journal.pone.0180414 (PMC5501525; doi:10.1371/journal.pone.0180414)
Supplement: S2 Fig — (DOC) [file pone.0180414.s002.doc]

| **Section/topic** | **#** | **Checklist item** | **Reported on page #** |
| --- | --- | --- | --- |
| **TITLE** | | |  |
| Title | 1 | Both. |  |
| **ABSTRACT** | | |  |
| Structured summary | 2 | Sorghum and millet grow in some of the most heterogeneous and austere agroecologies around the world. These two crops are amongst the top sources of food and feed crops and their significance will continue to grow especially with climate related heat and drought stress. Yet, few studies document the impact of sorghum and millet genetic enhancement. The Internal Rate of Return (ROR) is one of the most popular metrics used to measure the economic return on investment on agricultural research and development (R&D). The study conducted a meta-analysis of 59 sorghum and millet estimates. The average rate of return to sorghum and millet R&D investment is in the range of 54-76 percent per year. All of the reviewed studies computed social rather than private ROR because sorghum and millet technologies were developed using public funds originating from host country National Agricultural Research Systems (NARS) and international organizations such as the INTSORMIL CRSP, ICRISAT and others. Nearly three quarter of the studies focused only on sorghum (72 percent) and around one tenth of the studies (8 percent) dealt only with millet. Regression models are used to analyze the determinants of variations in the reported RORs. The ROR measure characteristics, analysist characteristics, research characteristics, and evaluation characteristics are used as control variables. Results show that ex-ante type and self-evaluated type of analyses are positively and significantly associated with the ROR estimates. Compared to estimates conducted by a university, results from international institutions and other mixed organizations provided significantly smaller estimates. Estimates conducted at national level also are significantly lower than those conducted at sub-national levels. The study also reconstructed modified internal rate of return (MIRR) for a sub-sample of the reported RORs following recent methods from the literature. These results show that the MIRR estimates are significantly smaller than the reported ROR estimates. | 1 |
| **INTRODUCTION** | | |  |
| Rationale | 3 | Few studies document the impact of sorghum and millet genetic enhancement. No specific global review of sorghum and millet exist in the literature. | 2 |
| Objectives | 4 | The current study is a systematic review and analysis of the economic impact of agricultural R &D investments on sorghum and millet. A review of the past studies of economic impacts of agricultural R&D investment on sorghum and millet will help provide an empirical support to the extent of the economic gains achieved from these two crops. | 2-3 |
| **METHODS** | | |  |
| Protocol and registration | 5 | Not applicable. | NA |
| Eligibility criteria | 6 | The main feature of selection pertaining to the reviewed studies involved whether studies presented rates of return on either sorghum, or millet or both. Since this is the first of its kind for sorghum and millet studies, there was no time period restriction. No restrictions on the language of report or the publication status of the report was also placed. | 5 |
| Information sources | 7 | To identify the studies for the review, a comprehensive search was compiled of all the available evidence on the returns to agricultural R&D investments on sorghum and millet. The search initiated with the review of reference sections of the latest publications available online or on print. Each of the references cited was reviewed for information on impact assessment studies on sorghum and millet. Based on this information, the relevant impact assessment studies on sorghum and millet are traced repeatedly until no more relevant reference citation is found on the reference sections in a systematic process that is generally referred to as “snowballing”. Online search engines such as Google scholar, economic literature databases such as EconLit, and JSTOR, Agricola etc. and the Consultative Group on International Agricultural Research (CGIAR)’s Standing Panel on Impact Assessment publications database were used for the search. Personal contacts via email and phone calls with some of the authors are also made to retrieve a few of the relevant studies. In total, 25 studies and 54 ROR point estimates were assembled and are listed in appendix 1. | 4 |
| Search | 8 | Example of Google Scholar search engine used:  Find articles with all of the words: rate of return impact assessment sorghum millet  Where my words occur : any where in the article  Authored by : anyone  Published in : anywhere  Dates between : anytime |  |
| Study selection | 9 | Figure 1. | 4, 35 |
| Data collection process | 10 | Not applicable. | NA |
| Data items | 11 | Data items for meta-regression was based on the ROR measure characteristics, analysist characteristics, research characteristics, and evaluation characteristics which were defined as determinants of ROR. More specifically, **Characteristics of the Rate of Return Measure (*m*):** *real or nominal; marginal or average* ROR; *ex-ante* or *ex-post; social* or *private;* **Analyst Characteristics (a):** *self-evaluation or not; published or not;* **Research Characteristics (r):** *fields of science* (*basic, applied, and extension); type of technology* *(yield enhancement, pest or disease control, crop management, extension); research organization; Scope of research;* **Evaluation Characteristics (e):** *economic surplus* *analysis; formal supply and demand model.* | 5-8 |
| Risk of bias in individual studies | 12 | Not applicable. | NA |
| Summary measures | 13 | The average rate of return to sorghum and millet R&D investment is in the range of 54-76 percent per year.  Results from regression models showed that ex-ante type and self-evaluated type of analyses are positively and significantly associated with the ROR estimates. Compared to estimates conducted by a university, results from international institutions and other mixed organizations provided significantly smaller estimates. Estimates conducted at national level also are significantly lower than those conducted at sub-national levels. Results from the reconstruction of the MIRR revealed that these estimates are significantly smaller than the reported ROR estimates. | 1, 3, 13-15, 30-33 |
| Synthesis of results | 14 | Not applicable. | NA |

Page 1 of 2

| **Section/topic** | **#** | **Checklist item** | **Reported on page #** |
| --- | --- | --- | --- |
| Risk of bias across studies | 15 | Not applicable. | NA |
| Additional analyses | 16 | Regression analysis models were used to relate the rate of return measure and its determinants. Additional analysis was done to reconstruct MIRR using a subsample of the studies. | 4-8; 8-10 |
| **RESULTS** | | |  |
| Study selection | 17 | Figure 1. | 34 |
| Study characteristics | 18 | The ROR measure characteristics, analysist characteristics, research characteristics, and evaluation characteristics are used as determinants. **Characteristics of the Rate of Return Measure (*m*):** *real or nominal; marginal or average* ROR; *ex-ante* or *ex-post; social* or *private;* **Analyst Characteristics (a):** *self-evaluation or not; published or not;* **Research Characteristics (r):** *fields of science* (*basic, applied, and extension); type of technology* *(yield enhancement, pest or disease control, crop management, extension); research organization; Scope of research;* **Evaluation Characteristics (e):** *economic surplus* *analysis; formal supply and demand model;* | 5-7 |
| Risk of bias within studies | 19 | Not available. | NA |
| Results of individual studies | 20 | Tables 6-9 | 30-33 |
| Synthesis of results | 21 | Results presented in the text. | 13-18 |
| Risk of bias across studies | 22 | Not applicable. | NA |
| Additional analysis | 23 | Tables 6-9 ; MIRR results presented. | 30-33; 17 |
| **DISCUSSION** | | |  |
| Summary of evidence | 24 | The average ROR to sorghum and millet R&D investment is in the range of 54-76 percent per year. All of the reviewed studies computed social rather than private RORs because sorghum and millet technologies were developed using public funds from host country National Agricultural Research Systems (NARS) and international partners such as the International Crops Research Institute for the Semi-Arid Tropics (ICRISAT), International Sorghum and Millet Collaborative Research Support Program (INTSORMIL CRSP), and others. Nearly three quarter of the studies focused only on sorghum (72 percent of the publications) and around one tenth of the studies (8 percent of the publications) dealt only with millet. Regression results show that ex-ante type and self-evaluated type of analyses are positively and significantly associated with the rate of return estimates. Compared to estimates conducted by a university, results from international institutions and other mixed organizations provided significantly smaller estimates. The study also reconstructed modified internal rate of return (MIRR) for a sub-sample of the reported RORs following recent methods from the literature (Alston et al, 2000). These results show that the MIRR estimates are considerably smaller than the ROR estimates. | 1,3, 18 |
| Limitations | 25 | It is important to note that the smaller number of reported ROR estimates limits the generalization of the regression results of the study. The lack of variation in some of the determinants of the RORs also resulted in exclusion of some important variables. | 18 |
| Conclusions | 26 | Historical returns on sorghum and millet R &D investments have been socially profitable. Future research may find helpful to expand the review of literature as more and more studies become available. | 18 |
| **FUNDING** | | |  |
| Funding | 27 | This study is made possible by the support of the American People provided to the Feed the Future Innovation Lab for Collaborative Research on Sorghum and Millet through the United States Agency for International Development (USAID). The contents are the sole responsibility of the authors and do not necessarily reflect the views of USAID or the United States Government. Program activities are funded by the United States Agency for International Development (USAID) under Cooperative Agreement No. AID-OAA-A-13-00047 |  |

*From:*  Moher D, Liberati A, Tetzlaff J, Altman DG, The PRISMA Group (2009). Preferred Reporting Items for Systematic Reviews and Meta-Analyses: The PRISMA Statement. PLoS Med 6(7): e1000097. doi:10.1371/journal.pmed1000097

For more information, visit: **www.prisma-statement.org**.

Page 2 of 2
