# Supplementary material for: Rates of return to sorghum and millet research investments: A meta-analysis
Source: PLoS One. 2017 Jul 7;12(7):e0180414. doi: 10.1371/journal.pone.0180414 (PMC5501525; doi:10.1371/journal.pone.0180414)
Supplement: S1 Table — (DOCX) [file pone.0180414.s003.docx]

**S1 Table. List of publications included in the meta-analysis.**

| No. | First Author (year of publication) | Internal Rates of returns | Geographical Region | Crop(s) |
| --- | --- | --- | --- | --- |
| 1 | Ahmed M.M. (1991) | 31 | Africa | Sorghum |
| 2 |  | 23 | Africa | Sorghum |
| 3 | Ahmed M.M. (1995) | 97 | Africa | Sorghum |
| 4 |  | 53 | Africa | Sorghum |
| 5 |  | 96 | Africa | Sorghum |
| 6 | Aghib, A.J. (1996) | 56 |  | Sorghum |
| 7 | Anandajayasekeram, P.(2007a) | 25 | Africa | Sorghum |
| 8 | Anandajayasekeram, P.(2007b) | 14 | Africa | Sorghum and Millet |
| 9 |  | 5 | Africa | Sorghum and Millet |
| 10 | Anandajayasekeram, P.(2007c) | 13.28 | Africa | Millet |
| 11 | Anandajayasekeram P,(1995) | 27 | Africa | Sorghum |
| 12 | Araji, A. A.( 1981) | 73.75 | United States | Sorghum |
| 13 |  | 74.42 | United States | Sorghum |
| 14 |  | 74.46 | United States | Sorghum |
| 15 |  | 112.88 | United States | Sorghum |
| 16 |  | 112.88 | United States | Sorghum |
| 17 |  | 113.06 | United States | Sorghum |
| 18 | Ayanwale, A.B. (2013) | 29 | Africa | Sorghum |
| 19 |  | 29 | Africa | Sorghum |
| 20 |  | 37 | Africa | Sorghum |
| 21 |  | 22 | Africa | Millet |
| 22 |  | 22 | Africa | Millet |
| 23 |  | 29 | Africa | Millet |
| 24 | Chisi, M.(2007)§ | 12 | Africa | Sorghum |
| 25 | Damte, T. (2009) | 329 | United States | Sorghum |
| 26 |  | 370 | United States | Sorghum |
| 27 |  | 389 | United States | Sorghum |
| 28 | Eddleman, B.R. (1991) | 48.2 | United States | Sorghum |
| 29 |  | 33.4 | United States | Sorghum |
| 30 |  | 41.7 | United States | Sorghum |
| 31 |  | 33.5 | United States | Sorghum |
| 32 | Gonzalez-Rey, D. (1991) | 32 | Central America | Sorghum |
| 33 | Griliches, Z. (1958) | 400 | United States | Sorghum |
| 34 | Kristijanson, P.M. (1999) | 28 | Asia | Sorghum and Millet |
| 35 |  | 38 | Asia | Sorghum and Millet |
| 36 |  | 43 | Asia | Sorghum and Millet |
| 37 | Mazzucato, V. (1994 ) | 21 | Africa | Sorghum and Millet |
| 38 |  | 2 | Africa | Sorghum and Millet |
| 39 | Otto, D.M.(1981) | 134.1 | United States | Sorghum |
| 40 |  | 93.8 | United States | Sorghum |
| 41 |  | 101.2 | United States | Sorghum |
| 42 |  | 116 | United States | Sorghum |
| 43 |  | 47 | United States | Sorghum |
| 44 |  | 79.5 | United States | Sorghum |
| 45 |  | 112.7 | United States | Sorghum |
| 46 |  | 133.5 | United States | Sorghum |
| 47 |  | 42.1 | United States | Sorghum |
| 48 |  | 63.2 | United States | Sorghum |
| 49 | Rohrbach, D.D. (1999) | 50 | Africa | Millet |
| 50 | Sanders , J.H. (1994) | 2 |  |  |
| 51 | Sterns, J. A. (1993) | 1 | Africa | Sorghum |
| 52 | Sterns, J.A.(1994) | 1 | Africa | Sorghum |
| 53 | Villacís A.H. (2011) | 37 | Central America | Sorghum |
| 54 | Yapi, A. (1998) | 95 | Africa | Sorghum |
| 55 | Yapi, A. (1999) | 95 | Africa | Sorghum |
| 56 |  | 75 | Africa | Sorghum |
| 57 |  | 71 | Africa | Sorghum |
| 58 | Yapi, A.M. (2000) | 69 | Africa | Sorghum |
| 59 |  | 50 | Africa | Millet |

Aghib, A.J., 1996. “Economic Impact Assessment of the World Vision International-Purdue University *Striga* Resistant Sorghum Initiatives”. Report prepared for World Vision International under USAID Award No. AOT-0000-G-00-5094-00.

Ahmed MM, Masters WA, Sanders JH .1995. “Returns from research in economies with policy distortions: Hybrid sorghum in Sudan.” *Agricultural Economics* 12 183-192.

Ahmed MM, Sanders JH .1991. “The Impact of Hageen Dura 1 in the Gezira Scheme, Sudan”. INTSORMIL Project: Nebraska, Lincoln.

Anandajayasekeram P, Martella DR, Sanders J, Kupfuma B .2007. Ex-ante analysis of the sorghum and millet improvement program. In 'Impact of Science on African Agriculture and Food Security '. (Eds P Anandajayasekeram, M Rukani, S Babu, F Liebenberg, CL Keswani) (CABI Publishing.: Cambridge, MA)

Anandajayasekeram P, Martella DR, Sanders J, Kupfuma B .2007. “Impact of pearl millet research and development in Namibia: the case of Okashana 1.” In P. Anandajayasekeram, M. Rukani, S. Babu, F. Liebenberg, and C.L. Keswani , eds. *Impact of Science on African Agriculture and Food Security*. CABI Publishing: Cambridge, MA

Anandajayasekeram P, Martella DR, Sanders J, Kupfuma B.2007. “Impact of sorghum research and development in Zimbabwe: the case of SV 2.” In P. Anandajayasekeram, M. Rukani, S. Babu, F. Liebenberg, and C.L. Keswani , eds. *Impact of Science on African Agriculture and Food Security*. CABI Publishing: Cambridge, MA

Anandajayasekeram, P., D.R. Martella, J.H. Sanders, and B. Kuffuma. "Report on the Impact Assessment of the SADAC/ICRISAT Sorghum and Millet Improvement Program." Southern African Development Coordination Conference (SADCC) and Southern African Center for Cooperation in Agricultural Research (SACCAR), Ganorone, Botswana, 1995. Mimeo.

Araji, A.A.1981. “The economic impact of investment in integrated pest management.” In G.W. Norton ed. *Evaluation of agricultural research*. Minnesota Agricultural Experiment Station: Minneapolis, Minnesota, U.S.A., pp. 121-139.

Ayanwale, A.B., Adekunle, A.A., Akinola, A. A. and Adeyemo,V.A. 2013. “Economic Impacts of Integrated Agricultural Research for Development (IAR4D) in the Sudan Savanna of Nigeria”, *African Development Review*, 25(1): 30–41

Chisi M .2007. “Impact Assessment of Sorghum Research in Zambia.” In P. Anandajayasekeram, M. Rukani, S. Babu, F. Liebenberg, and C.L. Keswani , eds. *Impact of Science on African Agriculture and Food Security*. CABI Publishing: Cambridge, MA

Damte T, Pendelton BB, Almas LK .2009. “Cost Benefit Analysis of Sorghum Midge, stenodiplosis sorghicola, (coquillett)-Resistant Sorghum hybrid Research and Development in Texas.” *South Western Entomologist* 34:395-405.

Eddleman BR, Chang CC, McCarl BA .1991. “Economic benefits from INTSORMIL grain sorghum variety improvements in the United States.” Texas Agricultural Experiment Station: Texas A&M University, College Station, TX.

Gonzalez-Rey D, Lopez-Pereira M, Sanders JH .1991. “The Impact of New Sorghum Cultivars and other Associated Technologies in Honduras.” INTSORMIL Project: Nebraska, Lincoln.

Griliches Z .1958. “Research Costs and Social Returns: Hybrid Corn and Related Innovations.” *Journal of Political Economy* 66: 419-431.

Kristijanson, P.M, and Zerbini E .1999. “Genetic enhancement of sorghum and millet residues fed to ruminants. An ex ante assessment of returns to research.” ILRI (International Livestock Research Institue): Nairobi, Kenya.

Mazzucato V. and L. Samba.1994. “An Economic Analysis of Research and Technology Transfer of Millet, Sorghum, and Cowpeas in Niger.” MICHIGAN STATE UNIVERSITY: East Lansing, Michigan.

Otto, D.M.1981. “An economic assessment of research and extension investments in corn, wheat, soybeans and sorghum” PhD Dissertation , Virginia Polytechnic Institute and State University

Rohrbach, D.D., W.R. Lechner, S.A. Ipinge, and E.S. Monyo .1999. “Impact from investments in crop breeding: the case of Okashana 1 in Namibia.” International Crops Research Institute for the Semi Arid Tropics.: Patancheru , Andhra Pradesh, India.

Sanders, J.H .1994. “Economic impact of the commodity research networks of SAFGRAD.” In Sanders, J.H., T.Bezuneh, and A.C. Schroeder.eds. *Impact assessment of the SAFGRAD Commodity Networks* USAID: Washington, DC.

Sterns, J.A. 1993. “Ex Post Assessment of Investments in Cameroon's Cowpea and Sorghum Research-Extension Systems.” MSc Thesis, Michigan State University.

Sterns, J.A, and R.H. Bernsten .1994. “Assessing the impact of cowpea and sorghum research and extension in Northern Cameroon.” Department of Agricultural Economics, Michigan State University: East Lansing, Michigan.

Villacís, A.H, and J.H. Sanders . 2011. “Returns to the Introduction of New Sorghum Cultivars into the Dairy Industry of El Salvador” Paper presented at CENTA conference “Cambio Climático e Impacto de los Sorgos Forrajeros.” San Salvador, El Salvador.

Yapi, A.M., A.O. Kergna, S.K. Debrah, A. Sidibe, and O. Sanogo .2000. “Analysis of the economic impact of sorghum and millet research in Mali.” International Crops Research Institute for the Semi-Arid Tropics: Andhra Pradesh, India.

Yapi, A., G. Dehala, K. Ngawara, and I. Abdallah .1998. “Sorghum S 35 in Chad — adoption and benefits.” In Bantilan,M.C.S. and Joshi, P.K.Eds. *Assessing joint research impacts: proceedings of an International Workshop on Joint Impact Assessment of NARS/ICRISAT Technologies for the Semi-Arid Tropics*. International Crops Research Institute for the Semi-Arid Tropics: Patancheru pp. 11-25., India

Yapi, A.M., S.K. Debrah, G. Dehala, and C. Njomaha .1999. “Impact of germplasm research spillovers: the case of sorghum variety S 35 in Cameroon and Chad.” International Crops Research Institute for the Semi Arid Tropics: Andhra Pradesh, India.
