# Supplementary material for: Rates of return to sorghum and millet research investments: A meta-analysis
Source: PLoS One. 2017 Jul 7;12(7):e0180414. doi: 10.1371/journal.pone.0180414 (PMC5501525; doi:10.1371/journal.pone.0180414)
Supplement: S2 Table — (DOCX) [file pone.0180414.s004.docx]

**S2 Table. Meta-analysis regression results for sorghum and millet ROR studies excluding very high ROR estimates.**

|  | ROR | ROR | ROR | ROR | ROR |
| --- | --- | --- | --- | --- | --- |
| Constant | 80.36*** | 73.67*** | 71.24** | -3.93 | 48.17*** |
|  | (4.89) | (4.75) | (2.63) | (0.37) | (3.49) |
| Ex-ante estimate | 37.94*** | 25.99 | -21.40 | 33.35** | -6.42 |
|  | (2.82) | (1.55) | (1.29) | (2.33) | (0.58) |
| *Analyst characteristics* |  |  |  |  |  |
| Self-evaluation | 106.10*** |  |  |  |  |
|  | (3.21) |  |  |  |  |
| International institution affiliation | -32.40* | -2.38 |  | 60.84*** |  |
|  | (1.73) | (0.10) |  | (4.05) |  |
| International and academic institution | -34.75* | -86.31*** | -137.32*** | -86.12*** | -130.23*** |
|  | (1.97) | (22.50) | (9.03) | (23.55) | (13.74) |
| Study published | -28.03 | -9.13 | 19.33 | -37.12** | 1.14 |
|  | (1.63) | (0.52) | (1.62) | (2.62) | (0.13) |
| *Research Characteristics* |  |  |  |  |  |
| ROR reporting period |  | 68.33*** | 72.99*** | 68.33*** | 68.33*** |
|  |  | (25.35) | (14.47) | (24.95) | (24.95) |
| Multinational scope | -42.71** | -31.09 | 88.82** | -38.25** | 46.04*** |
|  | (2.56) | (1.55) | (2.57) | (2.16) | (2.78) |
| National scope | -36.46** | -37.79** | -34.41 | -36.97** | -36.42** |
|  | (2.70) | (2.63) | (1.45) | (2.66) | (2.60) |
| Sub-Saharan Africa region |  |  | -13.98 |  |  |
|  |  |  | (1.30) |  |  |
| United States region |  |  |  | 98.06*** | 47.22*** |
|  |  |  |  | (6.39) | (4.12) |
| Sorghum only | 33.19*** | 33.89*** | 52.00*** | 33.46*** | 33.31*** |
|  | (4.10) | (3.98) | (5.70) | (4.02) | (4.38) |
| INTSORMIL or ICRISAT |  |  | -61.76*** |  | -44.31*** |
|  |  |  | (5.73) |  | (5.59) |
| *Evaluation Characteristics* |  |  |  |  |  |
| Pivotal supply shift | -3.31 | -31.96** | -8.42 | 73.23*** | 26.78** |
|  | (0.17) | (2.33) | (0.69) | (4.11) | (2.47) |
| Parallel supply shift | -78.03*** | -83.10*** | -53.75*** | -40.36** | -25.41 |
|  | (3.88) | (3.00) | (3.08) | (2.13) | (1.56) |
| *R*^2^ | 0.46 | 0.44 | 0.70 | 0.73 | 0.74 |
| *N* | 43 | 43 | 43 | 43 | 43 |

* Significant at the 90 percent confidence level; ** significant at the 95 percent confidence level; ***significant at the 99 percent confidence level.

**^** the median ROR reporting period is 1995. The reporting period takes the value of 1 if the ROR report is published on or after the year 1995, and 0 otherwise.

*±* the ‘pivotal supply shift’ assumes a linear in logarithms supply function and shifts proportionally, whereas the “parallel supply shift’ assumes linear supply function and shifts in parallel.
