# Supplementary material for: Rates of return to sorghum and millet research investments: A meta-analysis
Source: PLoS One. 2017 Jul 7;12(7):e0180414. doi: 10.1371/journal.pone.0180414 (PMC5501525; doi:10.1371/journal.pone.0180414)
Supplement: S3 Table — (DOCX) [file pone.0180414.s005.docx]

**S3 Table. Meta-analysis regression results for sorghum and millet ROR studies with log transformed RORs**

|  | log ROR | log ROR | log ROR | log ROR | log ROR |
| --- | --- | --- | --- | --- | --- |
| Constant | 3.69*** | 3.92*** | 3.71*** | 0.43 | 2.09** |
|  | (9.18) | (5.47) | (7.7) | (1.41) | (2.53) |
| Ex-ante estimate | 0.69** | -1.30** | 0.36 | 0.68*** | -0.30 |
|  | (2.24) | (2.06) | (1.11) | (2.83) | (-0.84) |
| *Analyst characteristics* |  |  |  |  |  |
| Self-evaluation | 1.44*** |  |  |  |  |
|  | (3.6) |  |  |  |  |
| International institution affiliation | -1.07 |  | -0.78 | 1.93*** |  |
|  | (-1.55) |  | (-0.97) | (-2.97) |  |
| International and academic institution | -1.13*** | -3.59*** | -2.09*** | -2.29*** | -3.39*** |
|  | (-3.02) | (-5.24) | (-12.52) | (-15.66) | (-6.84) |
| Study published | 0.23 | 1.24** | 0.46 | -0.76*** | 0.19 |
|  | (0.47) | (2.07) | (0.96) | (-3.21) | (0.55) |
| *Research Characteristics* |  |  |  |  |  |
| ROR reporting period**^** |  | 1.46*** | 1.20*** | 1.41*** | 1.36*** |
|  |  | (3.82) | (7.10) | (9.36) | (8.28) |
| Multinational scope | -1.11* | 3.02** | -0.83 | -1.20** | 0.58 |
|  | (-1.82 | (-2.25) | (-1.48) | (-2.35) | (0.69) |
| National scope | -0.63* | -0.82 | -0.66*** | -0.69*** | -0.78*** |
|  | (-1.82) | (-1.45) | (-2.83) | (-3.17) | (-3.18) |
| Africa region |  | -1.30*** |  |  |  |
|  |  | (-2.90) |  |  |  |
| United States region |  |  |  | 4.29*** | 2.98*** |
|  |  |  |  | (15.46) | (4.82) |
| Sorghum only | 0.61*** | 1.25*** | 0.53*** | 0.47*** | 0.22 |
|  | (2.75) | (3.09) | (3.43) | (3.02) | (0.83) |
| INTSORMIL or ICRISAT |  | -1.92*** |  |  | -0.98** |
|  |  | (-3.40) |  |  | (-2.62) |
| *Evaluation Characteristics±* |  |  |  |  |  |
| Pivotal supply shift | -0.25 | 0.60 | -0.69** | 3.85*** | 2.57*** |
|  | (-0.66) | (0.98) | (-2.56) | (13.05) | (4.38) |
| Parallel supply shift | -0.52 | -0.11 | -0.73 | 0.91 | 1.44** |
|  | (-0.95) | (-0.24) | (-0.92) | (-1.20) | (-2.23) |
| *R*^2^ | 0.46 | 0.79 | 0.45 | 0.91 | 0.89 |
| *N* | 47 | 46 | 46 | 46 | 46 |

* Significant at the 90 percent confidence level; ** significant at the 95 percent confidence level; ***significant at the 99 percent confidence level.

**^** the median ROR reporting period is 1995. The reporting period takes the value of 1 if the ROR report is published on or after the year 1995, and 0 otherwise.

*±* the ‘pivotal supply shift’ assumes a linear in logarithms supply function and shifts proportionally, whereas the “parallel supply shift’ assumes linear supply function and shifts in parallel.
